# Thermomechanical and Structural Analysis of Manufactured Composite Based on Polyamide and Aluminum Recycled Material

**DOI:** 10.3390/polym16192742

**Published:** 2024-09-27

**Authors:** Adam Gnatowski, Rafał Gołębski, Krystian Stachowiak, Jana Petrů, Jakub Měsíček

**Affiliations:** 1 Department of Technology and Automation, Czestochowa University of Technology, 42-200 Czestochowa, Poland; krystian.stachowiaki@gmail.com; 2 Department of Machining, Assembly and Engineering Metrology, Technical University of Ostrava, 70800 Ostrava, Czech Republic; jana.petru@vsb.cz (J.P.); jakub.mesicek@vsb.cz (J.M.)

**Keywords:** polymer composites, aluminum filler, machining, DSC and DMTA testing, roughness, surface integrity parameters

## Abstract

The paper presents an analysis of the filler’s effect on the machining process and on changes in the thermomechanical properties of polymer composites based on aluminum chips. Composite research samples with a polymer matrix in the form of polyamide 6 were made by the pressing method. Comparative studies were carried out on the changes in thermomechanical properties and structure of the obtained molders with different filler contents and different fractions after the machining process. In order to determine the changes in thermal and mechanical properties, analysis was carried out using the differential scanning calorimetry (DSC) method, thermal analysis of dynamic mechanical properties (DMTA) and a detailed stereometric analysis of the surface. After mechanical processing, roughness amplitude parameters and volumetric functional parameters were determined. In order to analyze the structure, tomographic examinations of the manufactured composite were conducted. In relation to the polymer matrix, a significant increase in the storage modulus of the composites was noted in the entire temperature range of the study. An increase in the enthalpy of melting of the matrix was noted in composites with a lower filler content and a shift in the melting range of the crystalline phase. Significant differences were noted in the study of the composite surfaces in the case of using fillers obtained after machining with different fractions. The dependencies of the functional and amplitude parameters of the surfaces after machining of composite samples prove the change in the functional properties of the surface. The use of aluminum chips in the composite significantly changed the surface geometry.

## 1. Introduction

Developments in the manufacturing technologies for polymeric materials, including the search for innovative synthesis and production methods for polymers with specific properties, has resulted in an expansion of their application areas [1].

Currently, technologies constitute a crucial role in the production of polymers and composites, the recycling processes, efficient synthesis and production of materials [2].

Polymer materials are replacing metal and ceramic materials, addressing issues such as corrosion, weight, flexibility, and other challenges. In this context, technologies for producing eco-innovative polymer materials are being developed [3].

The practical use of new materials requires knowledge of their processing methods, mechanical and thermal properties, and structure, as well as recognition of changes in these properties during operation. Due to a growing number of issues related to the processing of polymeric materials, including waste from recycling, the use of modern materials in the production of products is becoming crucial in addressing global environmental pollution. Today, the development of new materials with improved properties and easy processability is particularly urgent because it offers relief to the environment and increases the acceptance of plastic products. Polymer composites, which are increasingly used in modern structures, are gaining importance as materials replacing traditional raw resources in various industrial sectors [4,5]. Thanks to their unique properties, such as lightness, high mechanical strength and corrosion resistance, these materials are used in the production of technologically advanced components and everyday items [6,7].

The use of aluminum waste can also contribute to reducing the costs of composite production, because these materials are often available at lower prices than new raw materials. Research related to processing technology and the possibility of using aluminum waste as a filler in the process of producing composites on a polymer matrix is very important in the aspect of recycling waste materials and is conducted by the authors of many works. In the paper [8], research on the properties of composite materials produced by compression molding of a compound of aluminum flakes and nylon 6 powder was presented. The electrical conductivity, density, hardness and morphology of the composites were studied. It was shown that for certain sizes of filler particles, the hardness initially decreases with increasing aluminum content, probably due to poor surface contact with the nylon matrix, but starting from a certain value, the hardness increases. Similar research on the production and properties of a thermoplastic polymer composite with aluminum filler was conducted by Osman and Mariatti [9]. The paper presents the results of studies on polypropylene composites filled with aluminum with different shapes of filler particles and filler content up to 55%. The influence of the content and shape of filler particles on the changes in the properties of the composites produced was identified. Schricker et al. [10] studied the properties of polyamide 6 and aluminum in the laser joining process and assessed the mechanical properties of the joint. Material tests were carried out in different positions within the joining zone. Pinto et al. [11] developed a hybrid of aluminum chips and reinforced polystyrene composite material using a blending process followed by thermocompression as an experimental mixture design technique. The component materials used were studied using XRF and FTIR analyzers. Anis et al. [12] studied polyethylene terephthalate filled with micro and nano aluminum powder. Similarly to other composites containing low aspect ratio filler particles, they obtained a slight increase in the tensile modulus with loading. In work [13], the mechanical behavior of composites containing nickel microparticles and aluminum microparticles and nanoparticles, prepared by casting and curing with epoxy resin, was investigated to determine their potential use as structural energy materials. Bishayet al. [14] present test results for the electrical, mechanical and thermal properties of polyvinyl chloride composites filled with various amounts of aluminum powder up to 40% by weight. It was found that the mechanical strength values decrease with increasing aluminum content.

In the article [15], Suhas et al. described the process of manufacturing a polymer composite made of epoxy resin, aluminum and copper powder, using the open-mold vacuum technique. A new type of composite material was developed by combining copper and aluminum powders with filler content up to 20%. The mechanical properties of the composite were tested, including hardness, tensile strength, percentage elongation, bending and impact strength. The use of aluminum particles causes significant changes in the properties and structure of polymer blends. In the case of polybutylene terephthalate/polyethylene terephthalate mixtures with filler in the form of aluminum flakes, a significant increase in the nucleating capacity in the polymer structure was found, as well as a decrease in the susceptibility of polymers to degradation [16]. Dasture et al. [17] produced a composite material based on aluminum powder and polyethylene by compression molding. In compression technology, it is possible to obtain composites with a low content of polymer matrix, which is important from the point of view of recycling. The use of various technologies for producing composites based on recycled materials, such as aluminum, is also important [18]. It should also be noted that injection molding technology is increasingly used in the production of composite materials, and therefore, it is increasingly important to explain the relationship between processing, structure and properties in injection molding for this group of materials, what he noticed in his work Zhou et al. [19].

The processing of polymer composites is a demanding process and depends significantly on their material properties. Composites enriched with mineral fillers, reinforced with glass fibers and those showing high elasticity are groups of products that present unique challenges in the context of machining. Due to the poor thermal conductivity and relatively low melting point of most polymer composites, special attention should be paid to the appropriate selection of tools and process parameters in order to advantageously remove heat during machining. The mechanical properties of polymer composites in which aluminum alloy fillers are used have a significant impact on the machining process [20]. These fillers are introduced into the polymer matrix to improve the strength, stiffness, and thermal or electrical conductivity of the composite. However, these metal additives introduce certain challenges in the machining process [21]. An important aspect is the hardness of the fillers compared to the polymer matrix. Fillers are much harder than most polymers, which can lead to faster wear of cutting tools. As a result, the machining process for filled polymer composites can be more demanding, which increases the impact on production costs [22,23,24,25]. During machining, temperatures can increase, especially in the tool–material contact areas. Aluminum can contribute to thermal conductivity, which can lead to increased temperatures in the cutting zones. Excessive heat can cause material deformation, which negatively affects machining quality. Hardness, delamination, temperature control and tool wear are key issues to consider in machining composite materials with metal fillers. Optimization of machining parameters and selection of appropriate tools and machining strategies are essential to achieve the desired results [26,27].

In the process of machining composites, the phenomena that often occur have a peculiar character compared to the machining of metal materials [28]. During the machining of polymeric and composite materials, as a result of the forces acting on them, the surfaces after machining are subject to unfavorable degradation; in addition, the process may be accompanied by a number of undesirable phenomena related to the impact on the external environment [29]. This is particularly influenced by the properties of the material itself, such as its porosity, shape and grain size. These are factors that significantly affect the course of the machining process of such a material, as well as the quality of the machined surface. The aim of the research was to analyze the effect of chips from aluminum alloys as fillers in polymer composites on their thermal and mechanical properties and the machining process. The analysis included comparative studies of changes in the thermomechanical properties of the manufactured composites, taking into account changes in the structure of the surface after the machining process. The research aimed not only to assess the quality of the machined surfaces, but also the effect of recycling fillers on the efficiency of machining a composite based on thermoplastic material. The conducted research analysis in this area consisted in determining the influence of thermomechanical properties of the produced material on the course of the machining process while simultaneously indicating areas for proper selection of cutting parameters. The undertaken research topic is not only current, but also has important significance for industry and environmental protection.

## 2. Materials and Methods

**Figure 1:**
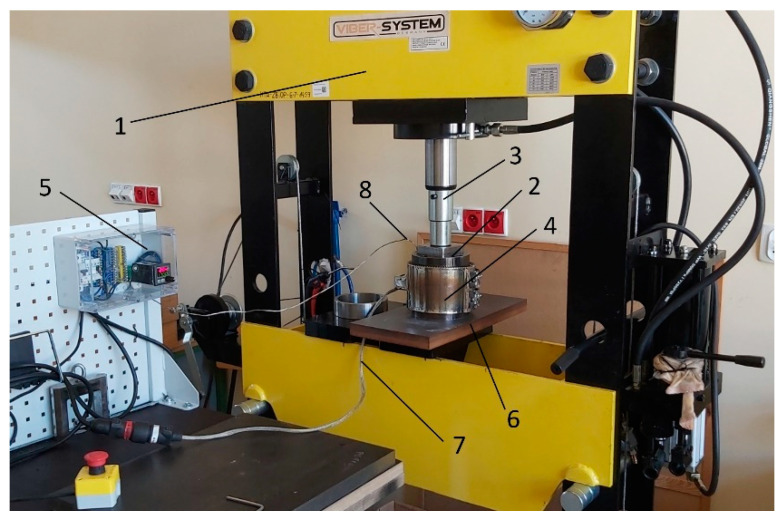
Laboratory stand for sample production: 1—hydraulic press, 2—form, 3—press piston rod, 4—band heating system, 5—temperature regulation system, 6—insulating plate, 7—power cord, 8—thermocouple.

In the process of manufacturing composites by pressing, the mutual percentage share and fraction of the matrix and filler have a very significant effect on the properties of the obtained material. The matrix was a polyamide PA6 with the trade name Alphalon^®^ 24 (Grupa Azoty S.A.) (Tarnow, Poland) obtained in the recycling process, fractionated in the grain size range up to 0.1 mm. The filler consisted of chips obtained as waste from the machining process of duralumin AW2017A, marked in accordance with the EN 573-1 standard AlCu4MgSi [30]. The chips were fractionated to obtain two fractions of 0.4–0.8 mm and 0.8–1.2 mm. The composite manufacturing process was carried out on the laboratory stand shown in Figure 1. The tool was a cylindrical pressing mold with a molding socket of dimensions 80 × 80 × 100 mm. The mold elements (socket, punch) were additionally subjected to a hardening process with high tempering, thanks to which their hardness was achieved at the level of 48 ± 2 HRC, which ensured the appropriate strength and durability of the tool during the multiple forming process.

### 2.1. Elaboration Process

**Figure 2:**
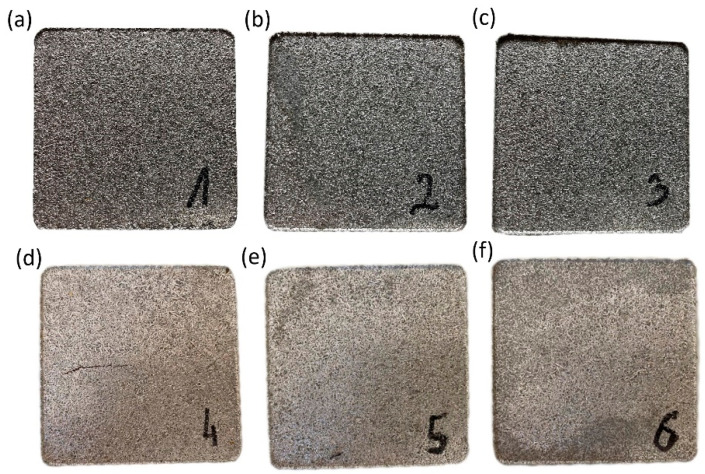
Samples produced with the following composition proportions: (**a**) PA5%/Al.95%, filler fraction 0.4–0.8 mm, (**b**) PA10%/Al.90%, filler fraction 0.4–0.8 mm, (**c**) PA15%/Al.85%, filler fraction 0.4–0.8 mm, (**d**) PA5%/Al.95%, filler fraction 0.8–1.2 mm, (**e**) PA10%/Al.80%, filler fraction 0.8–1.2 mm, (**f**) PA15%/Al.85%, filler fraction 0.8–1.2 mm.

The process of manufacturing composite samples with a filler in the form of aluminum chips consisted of several stages: first, a mixture of polymer with metal chips was prepared in the appropriate weight proportion for each sample, and the process of mixing the components was carried out in a drum mixer. Before the mixing process, the polymer material was dried at 80 °C for 12 h in a Shini CD-9-CE cabinet dryer (Shini Plastics Technologies, Inc., New Taipei, Taiwan). Then, the obtained mixture was poured into the mold cavity. In the next stage, the molding process was carried out in appropriate temperature and pressure conditions, which allowed obtaining the desired structure and mechanical properties of the composite. The mixture of PA6 material with chips placed in the mold cavity was homogenized at 230 °C under a pressure of 50 MPa using a hydraulic press. The heating system consisted of a cylindrical heating band with a power of 2400 W, together with a thermocouple and temperature stabilization system. The cooling process for the molded part was carried out at a pressure of 15.5 MPa and temperature ranging from 230 °C to 50 °C, for a duration dependent on the filler content, from 1500 s for the composite with 15% polymer matrix content to 2000 s for 10% polymer matrix content and up to 3000 s for 5% polymer matrix content. In order to maintain temperature stability, the mold was placed on a specially prepared insulating plate (6) made of high-pressure laminates (HPL) resistant to high temperature and constituting thermal insulation from the press table. The entire heating system, including the tool, was cooled with compressed air. In the conducted pressing process, 6 types of composite compacts were obtained in terms of composition—Figure 2, with dimensions of 80 × 80 × 10 mm, from which samples were prepared for testing thermomechanical and structural properties. The proportions of the composite composition were calculated according to the weight dependencies of the matrix and filler, taking into account the specific gravity of the materials. Table 1 lists the volumetric proportions.

### 2.2. Thermomechanical Characterization of Produced Composites

In order to assess the effect of changes in thermomechanical properties of the produced composites, due to the addition of filler in the form of aluminum chips, on the machining process, differential scanning calorimetry (DSC) and dynamic mechanical thermal analysis (DMTA) were performed. The DSC 214 Polyma device (Netzsch GmbH, Selb, Germany) was used to test thermal properties. Samples cut from the core of the molded parts were heated at a rate of 10 K/min in the temperature range of −30 to 280 °C. The measurements were carried out in a nitrogen atmosphere. As a result of the tests, thermograms of energy flux changes as a function of temperature were recorded. The glass transition temperature, crystalline phase melting temperature and melting enthalpy of the tested materials were determined using the DSC method. The tests were carried out in accordance with the PN-EN ISO 11357-3:2018-06 standard [31]. Thermal analysis of dynamic mechanical properties was performed in accordance with PN-EN ISO 6721-1:2019 [32] using a NETSCH DMA 242 C device (Netzsch Group, Selb, Germany) with a holder for three-point free bending of a sample in the form of a beam with dimensions of 50 × 10 × 4 mm cut out from composite moldings. The sample placed in the holder was subjected to the impact of a sinusoidally varying force with a frequency of 1 and 10 Hz and constant amplitude of 80 µm, while being heated at a rate of 3 °C/min from a temperature of −80 to 200 °C. Based on the determined values of force and deformation, taking into account the dimensions of the sample, the values of conservative modulus E’ and the tangent of the mechanical loss angle tgδ were calculated. In the analysis of DSC and DMTA test results, calculations were performed using Netzsch Proteus ver. 9.2.0 software (Netzsch Group, Selb, Germany).

### 2.3. Machinability of the Composites

**Figure 3:**
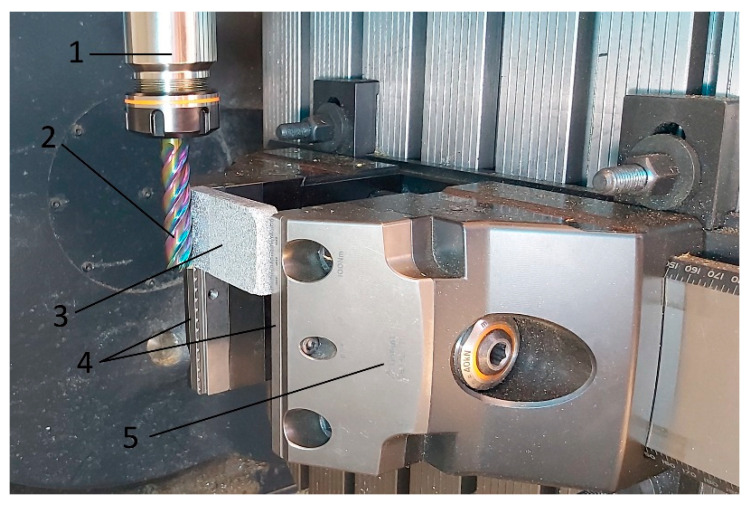
Composite machining process: 1—tool handle, 2—tool, 3—machined sample, 4—mounting jaws, 5—dedicated 5 axis vice.

Machining of composite materials with a non-uniform material structure is a great challenge in terms of selecting the right cutting tools and the appropriate technological parameters for the process. The milling machining process is determined by appropriately selected technological parameters such as cutting speed, depth and width of cut, tool parameters: rake angle, cutting edge radius, tool material and its coating. Additionally, the mechanical and thermal properties of polymer composites have a significant impact on the machining process. Due to poor thermal conductivity and relatively low melting points in most thermoplastics, care should be taken to ensure that the amount of heat generated during machining is transferred to the processed part to the smallest possible extent. In the case of thermoplastics, increasing the rake angle and reducing the cutting depth leads to a reduced effect on the degree of surface deformation after machining. Due to its thermal and mechanical properties, the material PA6 polyamide reacts to changes in processing parameters in a different way than metal materials or even other types of polymers, such as thermosets. An increase in cutting speed while simultaneously reducing the feed rate leads to an increase in the heat generated in the cutting zone, which may then lead to melting or surface thermal degradation. Higher cutting speeds, on the other hand, may facilitate the removal of chips from the processing area along with the generated heat, which is beneficial for maintaining the quality of processing. However, in the case of some thermoplastics, soft and sticky chips may stick to the cutting tool blade. To avoid such a situation for the processing of composite materials, it is necessary to develop an appropriate strategy for selecting cutting parameters and technology in order to obtain the lowest possible process effect on the surface condition after processing. The machining was carried out on a DMG MORI CMX50U numerically controlled milling machine (DMG MORI, Famot, Pleszew, Poland). The processed materials, samples with dimensions of 80 × 40 × 10 mm, were clamped in the claw jaws of a vice dedicated to multi-axis machining—see Figure 3. During clamping, special attention was paid to the jaw clamping force, which did not exceed 30 kN in order to eliminate the occurrence of internal stresses in the material during machining. A solid carbide milling cutter with a diameter of 16 mm and an unequal spacing helix angle of 50 deg—with five blades—was used for cutting. The tool with a DLC coating is used for machining brass, aluminum, and also polymer materials (materials that give short or long chips during machining). The assumed processing parameters were feed rate per tooth fz = 0.05 mm/tooth, cutting speed Vc = 550 m/min, cutting depth ap = 25 mm, cutting contact width ae = 1 mm.

The tool shank was made in accordance with DIN 6535 HA with h5 tolerance. A tool holder made in accordance with ISO 7388-1 [33] type ER32 SK40 A100 was used, maintaining the concentricity accuracy of ≤3 µm and the balancing accuracy of G 2.5 at the rotational speed of 25,000 min^−1^ [34].

### 2.4. Surface Stereometry Investigation

**Figure 4:**
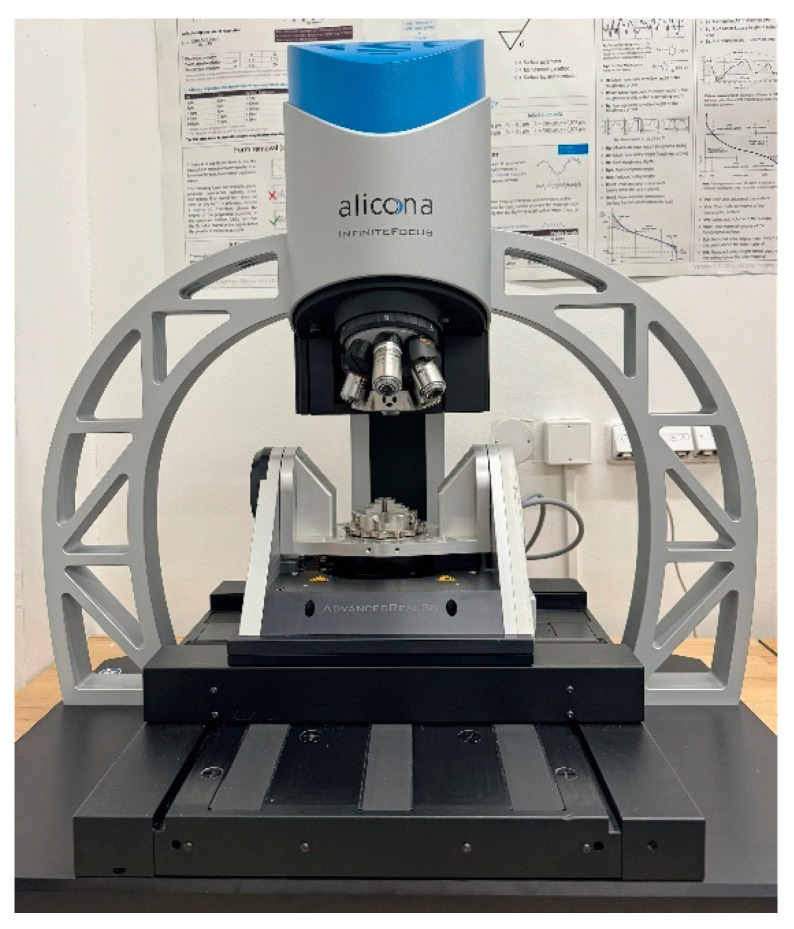
Alicona InfiniteFocus G5, surface integrity measuring microscope.

The surfaces of the machined samples were analyzed using an Alicona Infinite Focus optical microscope (Alicona Imaging GmbH, Raaba, Austria)—Figure 4. The measurement system operates on the principle of changing focus, using a surface scanning methodology that integrates the shallow depth of field of the optical system with vertical scanning, extracting topographic details in different focal planes. The method uses adjustable, white, concentric illumination directed at the sample. The sample surface reflects light rays, and their reflection is recorded by a sensor placed in the optical part of the microscope. Manipulating the sensor along the Z-axis changes the distance from the measured surface, thus adjusting the focal planes.

The study was conducted in accordance with the ISO 25178 [35] standard, determining, among others, roughness amplitude parameters (Sq—root mean square height, Sp—maximum peak height, Sv—maximum pit height, Sa—arithmetic mean height, Sz—maximum profile height, Sku—kutrose) and functional parameters (Vmc—core volume, Vvc—maximum pit volume, Vmp—peak volume, Vvv—percentage functional volume). Surface amplitude parameters describe the surface roughness profile, i.e., deviations of the actual surface from the ideally smooth one. These parameters provide important information on the surface structure, which is crucial in processes such as friction, wear or adhesion. In the analysis of surface structure, especially in the context of tribology and material technology, the evaluation of functional parameters Vmc, Vvc, Vmp and Vvv plays a key role in understanding the surface properties. These parameters allow for the evaluation of the surface characteristics in terms of its potential applications and performance properties. The core volume (Vmc) is a volumetric parameter that refers to the volume of surface material located in the core region, i.e., the central part of the surface profile that does not contain extreme peaks or deep valleys. The Vmc parameter is a measure of the structural stability of the surface and a key indicator for assessing the durability of the surface in mechanical applications. A high Vmc value indicates a more stable and durable surface, which is desirable in components exposed to high loads. The volume of the valleys (Vvc) is a parameter that refers to the volume of space between the surface and the reference line in the valley areas of the surface profile. It is a measure of the amount of free space that can be used, for example, to store lubricants or other substances. A high Vvc value indicates the ability of the surface to store lubricant effectively, which can significantly reduce friction and wear of mechanical components. The volume of the peaks (Vmp) is a parameter that refers to the volume of surface material located above the reference line in the peak areas of the surface profile. The Vmp value is a measure of the presence of peaks on the surface, which can affect initial contact and interactions with other surfaces. A low Vmp value indicates a smooth surface with few peaks, which can lead to reduced wear at the beginning of service. The percentage of the functional volume (Vvv) is a parameter that refers to the percentage of the valley volume in the total surface volume. It is an indicator of the surface’s effectiveness in terms of its ability to perform functional functions, such as storing lubricant or removing contaminants. A high Vvv value suggests that the surface has a well-developed valley structure, which may improve its ability to retain lubricant and remove contaminants, and thus increase the durability and reliability of the surface in long-term service.

### 2.5. Composite Amorphousness Investigation

**Figure 5:**
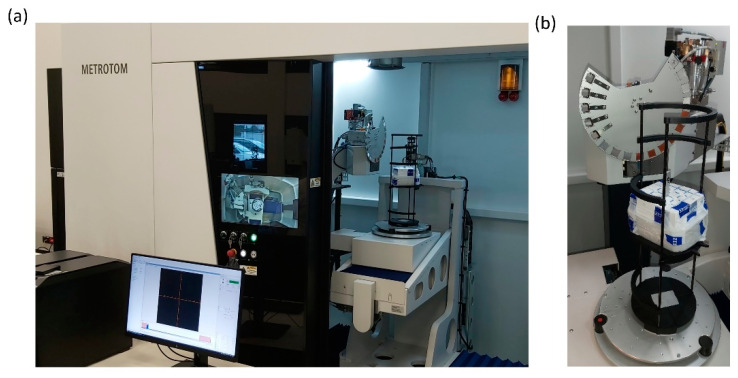
ZEISS Metrotom 1500 third generation tomograph: (**a**) device, (**b**) sample mounting system.

Computed tomography examinations are a type of X-ray spectroscopy, thanks to which tomographic images of the examined object are obtained, and then, thanks to the use of computer techniques for processing digital images, a spatial model (3D) is presented from many flat shots (2D) taken in different positions [36]. Tomographic images contain information about the position and absorbing features in the object and can be used for further reconstruction of spatial data. Any defects inside the object or changes in its density or porosity can be imaged and measured. The examination consists in directing an X-ray beam at the examined object and recording its intensity on the other side on the detector. X-ray radiation, similarly to electromagnetic radiation, is absorbed and scattered by the examined material depending on its properties. As a result of the material’s own absorption, the radiation beam is weakened. The weakening is closely related to the function of the radiation energy and the type and thickness of the examined material. The change in the radiation intensity of parallel beams of equal energy when passing through an object can be precisely defined by assigning specific features to particular types of material volumes. The study was performed using a Metrotom 1500 G3 computer tomograph (Carl Zeiss In-dustrielle Messtechnik GmbH, Oberkochen, Germany). The device is equipped with an advanced closed-type, water-cooled X-ray tube with a voltage range from 30 to 225 kV, which allows for the penetration of materials with higher density. The maximum power consumption of the tube is 500 W, which translates into higher efficiency and scanning speed—Figure 5. The detection system is based on an advanced high-resolution detector matrix, placed at a distance of up to 1500 mm from the X-ray source, which allows for obtaining detailed images with high precision. The size of a single pixel is 139 × 139 μm, which ensures exceptional image quality and measurement accuracy.

The samples were cut into 10 × 10 × 80 mm cuboids. The high-resolution 3 k detector provided detailed images of the composite samples with a voxel resolution of 0.034342 mm. The test samples were mounted in a low-density holder—see Figure 5b, which ensured stability during scanning. The low-density holder has a low X-ray absorption coefficient, which means that it does not cause significant artifacts on tomographic images. This allowed for obtaining cleaner and more precise images of the sample’s internal structure.

## 3. Results and Discussion

### 3.1. Thermal Analysis of Dynamic Mechanical Properties of the Produced Composites

**Figure 6:**
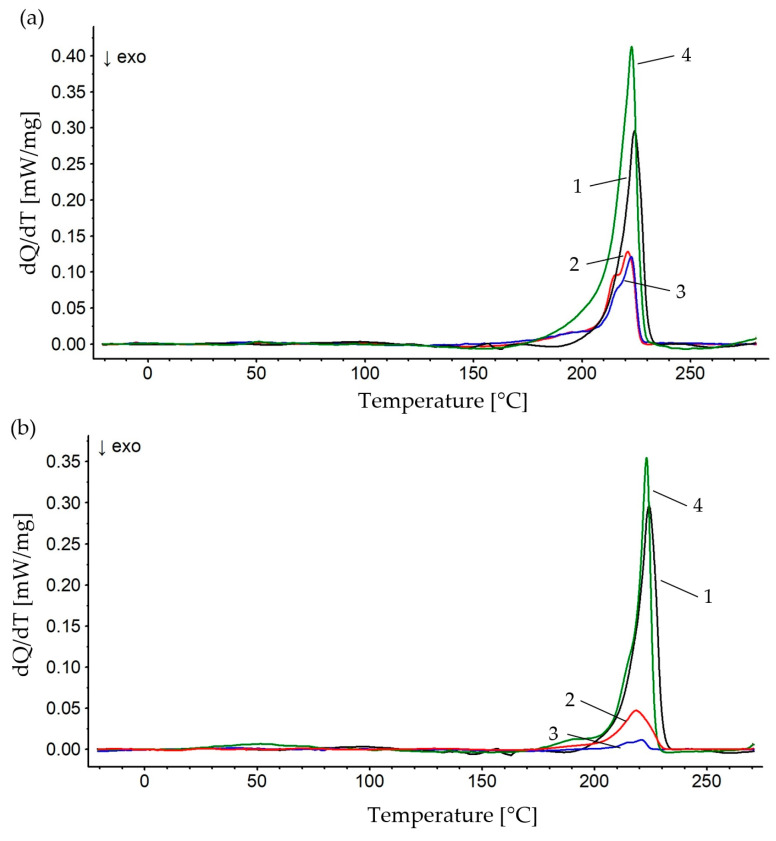
DSC thermograms of the tested materials: 1—PA6, 2—PA6 + 95% Al, 3—PA6 + 90% Al, 4—PA6 + 85%; (**a**) AL 0.4–0.8 mm, (**b**) AL 0.8–1.2 mm.

The obtained test results were analyzed in terms of chip fraction and different percentage of the matrix. Figure 6 shows thermograms of samples made of aluminum chips with fractions of 0.4–0.8 mm Figure 6a and 0.8–1.2 mm Figure 6b.

The analysis of thermograms and the test results presented in Table 2 indicate that the values of the temperature—the maximum melting reflex of the crystalline phase—was similar for all samples and fell within the range of 220.9–223.5 °C. The highest melting enthalpy values were recorded for composite samples with a higher polymer content, which indicates a higher degree of crystallinity of the polymer matrix. For samples with an Al content of 85% and a larger chip fraction, the AL fraction of 0.8–1.2 mm, a value of 10.86% was obtained, while for the fraction of 0.4–0.8 mm, a value of 18.28% was recorded. Considering the finely spherulitic structure of PA6 [37], the reason for the increase in the degree of crystallinity of the polymer matrix of the composite with 85% filler content is probably the possibility of additional formation of crystallization nuclei within the chips, which is smaller in the case of a larger chip surface of the fraction of 0.8–1.2 mm. These samples may be characterized by a more ordered structure, which significantly affects the stiffness of the material and mechanical parameters [38].

Higher content of chips in the composite results in blocking the possibility of crystalline phase growth in the technological process of composite production during cooling. These values are lower for samples with a higher filler content, which indicates different mechanical parameters and a more amorphous structure. The glass transition temperature for composite samples differs significantly. The lowest value of the glass transition temperature was noted for the sample with the highest content of polymer matrix. Immaculate PA6 and PA6 + 95% Al composite samples showed higher values for the glass transition temperature. Differences in the range of melting temperature, glass transition temperature and degree of crystallinity indicated diversity in the thermal structure stability of the samples.

**Figure 7:**
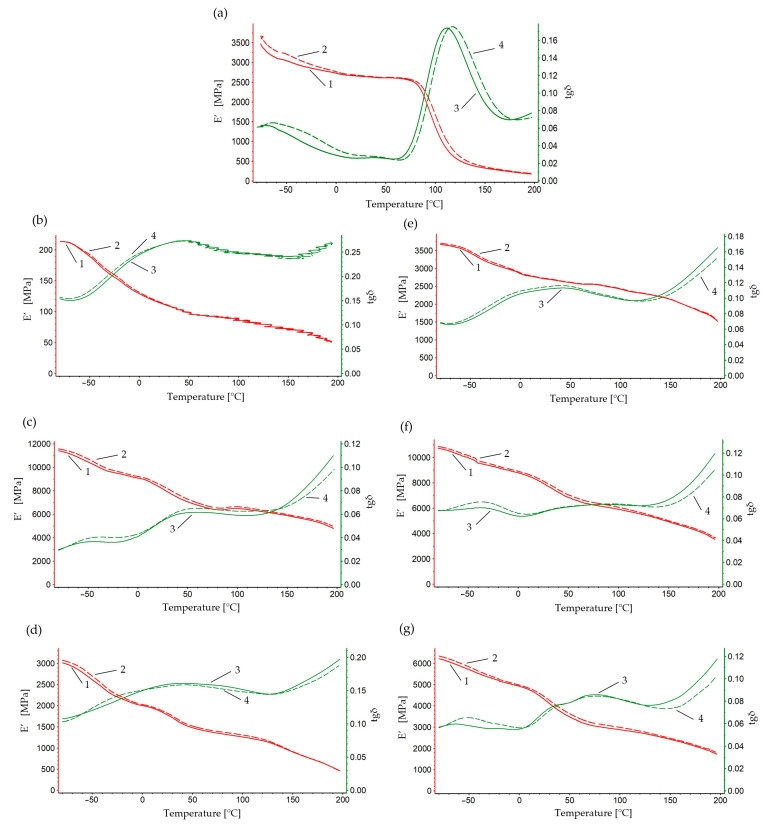
The dependence of the conservative modulus and the tangent of the mechanical loss angle on the temperature at a frequency of 1 Hz-1, 3, at a frequency of 10 Hz-2, 4: (**a**) PA 6, (**b**) PA6, 95% Al 0.4–0.8 mm, (**c**) PA6, 90% Al 0.4–0.8 mm, (**d**) PA6, 85% Al 0.4–0.8 mm, (**e**) PA6, 95% Al 0.8–1.2 mm, (**f**) PA6, 90% Al 0.8–1.2 mm, (**g**) PA6, 85% Al 0.8–1.2 mm.

Figure 7 shows the changes in the storage modulus and loss tangent as a function of temperature and vibration frequency for a sample made of polyamide 6. At the temperature of −80 °C, the highest E’ value was recorded, equal to 3480 MPa for 1 Hz and 3560 MPa for 10 Hz. The E’ modulus for PA6 decreases with increasing temperature, which indicates a decrease in the stiffness of the material at higher temperatures. The E’ value at the glass transition temperature was 2600 MPa. The glassy phase was recorded in the temperature range from 60 °C to 130 °C. This is a typical process for polymers, which change from the glassy phase to the viscoelastic phase. At the higher frequency of 10 Hz, higher values of the storage modulus (E’) were recorded than at the lower frequency of 1 Hz in the entire temperature range of the study, which indicates that the material is stiffer at faster loading cycles. In the high-elastic deformation phase, a significant decrease in the storage modulus value was noted. At the flow temperature of 175 °C, the E’ value was 255 MPa. The maximum recorded on the tan δ curve at a lower frequency of the acting force corresponds to the glass transition temperature. At 60 °C, the maximum tgδ value of 0.025 was recorded for both frequencies of force application. The maximum difference in the mechanical loss tangent was tgδ 0.01 for the applied frequencies. DMTA analysis for composites containing PA6 and aluminum chips showed significant changes in the values of the storage modulus E’ and the mechanical loss tangent tgδ compared to PA6. The values of the storage modulus E′ for the composite with chips of fraction 0.4–0.8 mm showed characteristic changes in the range of the test temperature: glassy phase, passing through the glass transition temperature Tg and the phase of highly elastic and plastic deformations. The highest E’ values of 11,580 MPa for 1 Hz and 11,710 MPa for 10 Hz were recorded for the PA10%/Al.90% sample with filler fraction 0.4–0.8 mm at a temperature of −80 °C. The difference in values compared to the PA6 sample was 8100 MPa for both frequencies of force application. In the entire temperature range, a difference of about 90 MPa was recorded at a temperature ranging from −80 °C to 200 °C. The value of the storage modulus at the glass transition temperature (78.5 °C) was 6320 MPa for a frequency of 1 Hz. In the analysis of the changes in the mechanical loss tangent from −80 °C to 0 °C, a similar course of the thermographic curves was recorded, and the difference in the mechanical loss modulus values between the frequencies of 1 Hz and 10 Hz was insignificant. In the case of the PA15%/Al.85% sample with filler fraction 0.4–0.8, the highest E’ values of 3040 MPa for 1 Hz and 3100 MPa for 10 Hz were recorded. For the composite, lower storage modulus values were recorded compared to the PA6 sample and amounted to 440 MPa for 1 Hz and 500 MPa for 10 Hz. The storage modulus value at the glass transition temperature (56.4 °C) was 1500 MPa for 1 Hz. On the other hand, for the PA5%/Al.95% sample with filler fraction 0.4–0.8, the E’ modulus value of 240 MPa was recorded at a temperature of −80 °C and frequency of 1 Hz, which means a decrease of 93% compared to PA6. The analysis of the test results for samples with a chip fraction of 0.8–1.2 mm confirmed that the E’ modulus values recorded in the test temperature range remained relatively high compared to PA6. For example, in the glassy phase at a temperature of −80 °C for the PA10%/Al.90% sample with filler fraction 0.8–1.2 mm, the maximum values of the E’ modulus were recorded at 1 Hz and 10,930 MPa at 10 Hz. At the glass transition temperature (75 °C), the E’ value was 6420 MPa at 1 Hz. The difference in the storage modulus value relative to the PA6 sample was 7310 MPa at 1 Hz and 7340 MPa at 10 Hz. In turn, the difference in E’ relative to the PA10%/Al.90% sample with filler fraction 0.4–0.8 mm was 800 MPa at 1 Hz and 770 MPa at 10 Hz at a temperature of −80 °C. For the PA5%/Al.95% sample with filler fraction 0.8–1.2 mm, the highest E’ value was recorded, equal to 3680 MPa for 1 Hz and 3720 MPa for 10 Hz at a temperature of –80 °C. A difference of about 50 MPa was noted in the entire temperature range.

The difference in the storage modulus value compared to the PA6 sample was 210 MPa for 1 Hz and 140 MPa for 10 Hz. At the glass transition temperature of 90 °C, the E’ value was 2500 MPa. For the composite sample PA15%/Al.85% with filler fraction 0.8–1.2 mm, the highest E’ value was recorded, equal to 6240 MPa for 1 Hz and 6370 MPa for 10 Hz at a temperature of −80 °C. The difference in the storage modulus value with respect to the PA6 sample was 2760 MPa for 1 Hz and 2770 MPa for 10 Hz at a temperature of −80 °C. A difference of about 100 MPa was noted in the entire range. At the glass transition temperature of 68.1 °C, the E’ value was 3050 MPa for 1 Hz. DMTA analysis showed that the addition of aluminum chips significantly affects the mechanical properties of PA6 composites. Depending on the fraction and content of chips, significant differences were recorded in the mechanical parameters of composite samples, which has a significant impact on the machining process for the produced materials.

### 3.2. Analysis of Surface Stereometry after Processing

**Figure 8:**
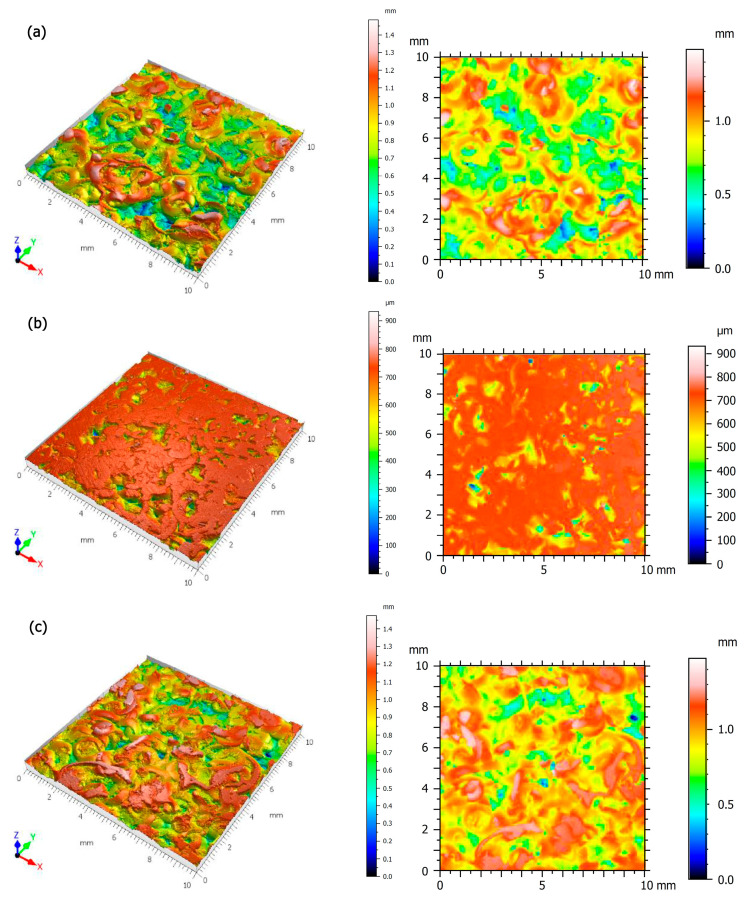

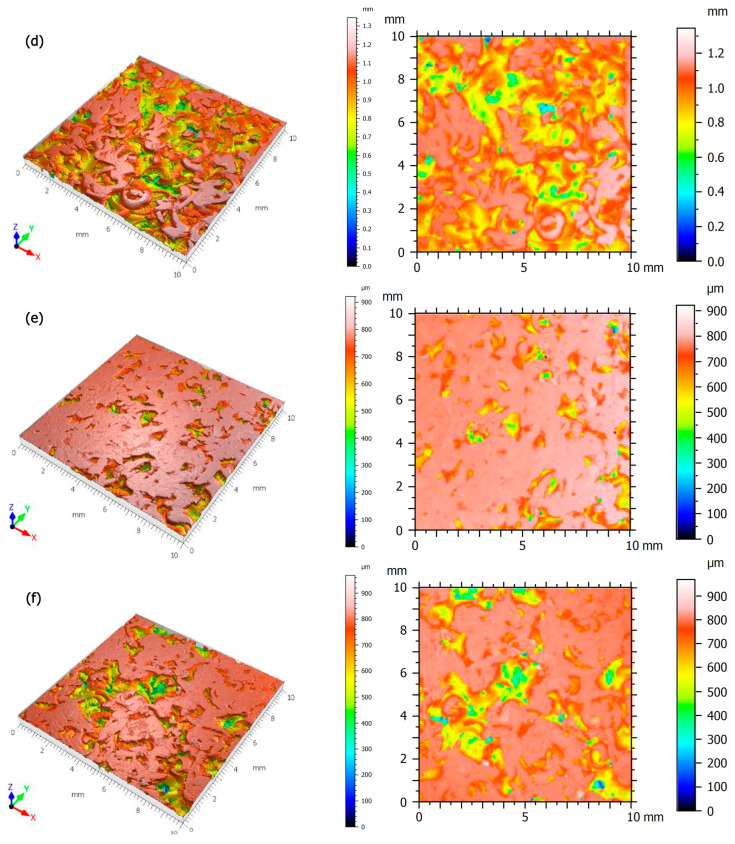
Stereometric visualization of the machined surfaces: (**a**) PA5%/Al.95%, filler fraction 0.4–0.8 mm, (**b**) PA10%/Al.90%, filler fraction 0.4–0.8 mm, (**c**) PA15%/Al.85%, filler fraction 0.4–0.8 mm, (**d**) PA5%/Al.95%, filler fraction 0.8–1.2 mm, (**e**) PA10%/Al.80%, filler fraction 0.8–1.2 mm, (**f**) PA15%/Al.85%, filler fraction 0.8–1.2 mm.

**Figure 9:**
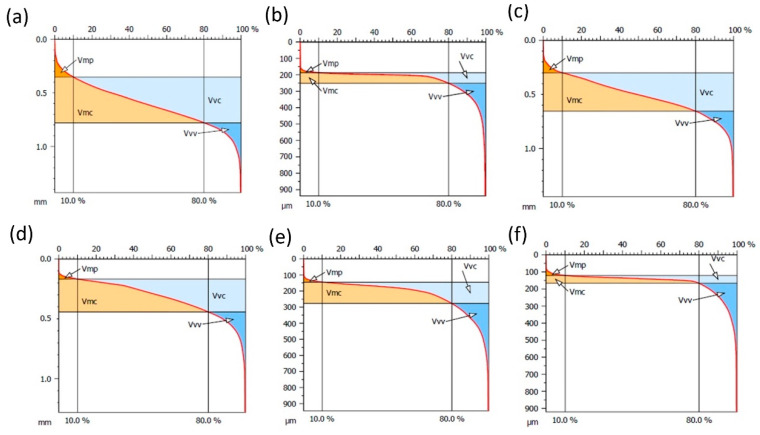
Graphic interpretation and volumetric parameters of machined composite surfaces: (**a**) PA5%/Al.95%, filler fraction 0.4–0.8 mm, (**b**) PA10%/Al.90%, filler fraction 0.4–0.8 mm, (**c**) PA15%/Al.85%, filler fraction 0.4–0.8 mm, (**d**) PA5%/Al.95%, filler fraction 0.8–1.2 mm, (**e**) PA10%/Al.80%, filler fraction 0.8–1.2 mm, (**f**) PA15%/Al.85%, filler fraction 0.8–1.2 mm.

The microgeometrical structure of the surface layer, in the central section of the cutting area, perpendicular to the machining direction, was assessed. Figure 8 shows the original structure of the stereometric distribution of the asperities of the machined composite samples. The material share curve analysis, known as the Abbott–Firestone curve, is also very often used in the analysis of surface topography. It provides important information on the surface characteristics, such as roughness, load-bearing capacity, fluid retention and wear resistance. It is interpreted as the percentage increase in the share of individual topography points in creating the total surface. The parameters determined from the material share curve enable a detailed analysis of the surface peaks and depressions, which is crucial for assessing its functionality and durability (Table 3). Figure 9 shows the material share curve for all samples. The aim of the study was to determine the influence of the fraction of aluminum chips and the proportion of PA6 material in the composite on the surface properties of this material. The amplitude and functional parameters were analyzed. The measurement strategy was implemented transversely to the machining direction, which is important in the interpretation of amplitude parameters.

### 3.3. Composite Amorphousness Analysis Using Computed Tomography—CT

As a result of the tomographic analysis, detailed 3D graphic records of the tested samples were obtained. Based on the preliminary analyses, the voxel size of 0.034342 mm was assumed. This assumption allowed for scanning the full volume of the sample in about 40 min. The volumetric analysis of the obtained results was performed using the dedicated ZEISS Inspect 2023, X-Ray software. In the research assumptions, the software defined and assigned the color to the imaged material, respectively: red—polymer matrix, blue—metallic filler, white—voids, black—contamination. A detailed visual analysis of the continuity of the sample cross-sections was performed, assuming the levels of composition homogeneity (degree of mixing materials), respectively: very high, high, moderate, low, very low. For the sample volume of 10 × 10 × 80 mm, the percentages of metallic filler, polymer matrix, voids, and contamination (undefined artefacts) were calculated using the software tool. The results of volumetric calculations are presented in Table 4.

In the analysis of the volumetric composition proportions, it is worth emphasizing the fact that material inhomogeneities in the form of inclusions—impurities—were recorded in all samples. The material obtained for testing came from recycling; the small amount of contamination confirmed the good quality of the matrix and filler components and did not in any way affect the changes in the material properties. The most undesirable feature was the occurrence of voids inside the material. The material used in the pressing process, under the action of force during the plasticization process, should uniformly penetrate the entire space inside, within the filler, which comprises chips of irregular shape. This process is strictly dependent on the filler fraction and the degree of the matrix–filler proportion.

**Figure 10:**
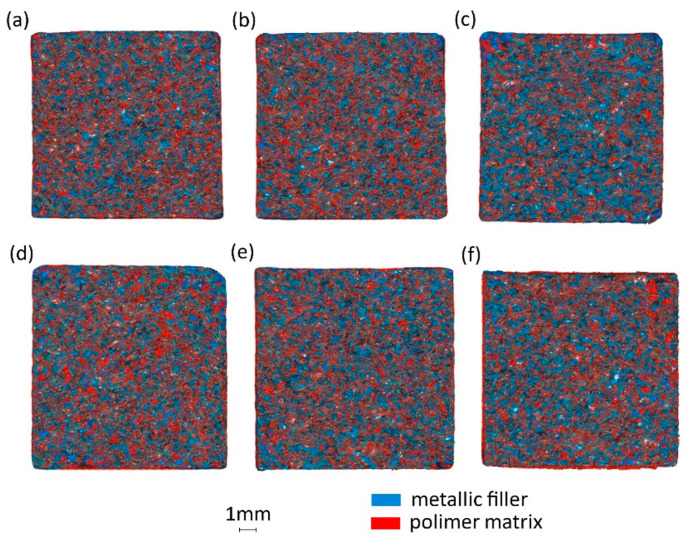
Cross-sectional images of samples in tomographic examination: (**a**) PA5%/Al.95%, filler fraction 0.4–0.8 mm, (**b**) PA10%/Al.90%, filler fraction 0.4–0.8 mm, (**c**) PA15%/Al.85%, filler fraction 0.4–0.8 mm, (**d**) PA5%/Al.95%, filler fraction 0.8–1.2 mm, (**e**) PA10%/Al.80%, filler fraction 0.8–1.2 mm, (**f**) PA15%/Al.85%, filler fraction 0.8–1.2 mm.

In the case of samples Figure 10c,f with the highest percentage of matrix, its excessive transmission to the tool walls occurred, generating voids inside the material due to the large volumes of gases. Such behavior may indicate that the percentage composition of matrix at the level of 15% is definitely too high. We can also see that the size of the filler fraction significantly affected the uniformity of the binder presence in the composite. In the case of samples with a polymer filling in the volume of 15%, there was a loss of material in the sample volume, which was caused by the formation of edge flashes during the pressing process. Analyzing the cross-sectional image of samples—see Figure 10, the highest degree of uniformity of composition was noticeable for the filler fraction of 0.4–0.8 mm, with particular attention paid to sample Figure 10b PA10%/Al.90%.

## 4. Conclusions

The aim of the conducted research was to analyze specific thermomechanical and structural properties of the produced composite material dedicated to final shaping by machining. In the work, special attention was paid to the composite production process in the research context of the proportions of its composition and the size of the filler fraction. The research aimed not only to understand the fundamental aspects of the composite behavior under conditions of temperature and load changes, but also to provide practical guidelines for engineers involved in the creation of composite materials subjected to the machining process.

Analyzing the research results, the following key conclusions were drawn:

- Increasing the content of metallic filler improved the mechanical properties of composites; however, an excessive amount of filler may lead to weakening of the material elasticity and an increase in its brittleness.
- The filler fraction (metal chip size) significantly affected the internal structure of the composites. Samples with a smaller chip fraction showed better structural homogeneity and lower porosity, which directly translated into improved mechanical properties.
- The material with a higher aluminum content was characterized by a higher glass transition temperature (Tg) and greater mechanical stability at high temperatures.
- The use of a larger filler fraction led to the formation of uneven surfaces after processing, which resulted in higher values for the cavity volume and larger surface defects.
- The even distribution of chips in the polymer matrix allowed for minimization of surface defects, such as pores or chipping, which could occur during machining.
- The use of a smaller filler fraction led to a more homogeneous internal structure, which resulted in reduced porosity of the composites and directly translated into better mechanical properties and resistance to dynamic loads.

The test results indicate the need to optimize the composition of composites depending on the intended application. In applications requiring high mechanical strength and minimal porosity, it is recommended to use a higher matrix content (15%) and smaller chip fractions. In applications where the priority is to obtain specific surface properties, such as increased roughness to improve adhesion, composites with larger chips and less matrix may be the appropriate choice.

## Figures and Tables

**Table 1:** The volumetric and weight composition of the samples.

Type of Material	Material Weight in Sample [g]	Material Weight Share in Sample [%]	Specific Weight of Material [g/cm^3^]	Material Volumetric Share in Sample [%]
Polyamide 6	7.5	5	1.13	11.50
Polyamide 6	15	10	1.13	21.53
Polyamide 6	22.5	15	1.13	30.35
Duralumin AW2017A	142.5	5	2.79	88.50
Duralumin AW2017A	135	10	2.79	78.47
Duralumin AW2017A	127.5	15	2.79	69.65

**Table 2:** Comparison of differential scanning calorimetry results obtained from Netzsch Proteus calculations.

Type of Material	Dhip Fraction [mm]	Max. Melt Temperature [°C]	Melting Range [°C]	Enthalpy, [J/g]	Degree of Crystallinity [%]	Temp. Glass Transition, [°C]
PA6		223.6	212.4–226.2	18.94	9.96	81.2
PA6 + 95% Al	0.4–0.8	220.9	208.0–223.3	12.51	6.58	83.4
PA6 + 90% Al	0.4–0.8	223.2	199.5–226.2	11.08	5.83	78.5
PA6 + 85% Al	0.4–0.8	222.4	218.4–226.7	34.74	18.28	56.4
PA6 + 95% Al	0.8–1.2	221.7	212.7–225.6	5.219	2.74	90.9
PA6 + 90% Al	0.8–1.2	222.7	214.1–224.5	1.014	0.57	75.6
PA6 + 85% Al	0.8–1.2	223.5	221.6–226.1	20.64	10.86	68.1

**Table 3:** Functional parameters of samples with PA6 and aluminum chips with fractions of 0.4–0.8 mm and 0.8–1.2 mm.

Type of Composite Sample	Vmp [ml/m^2^]	Vmc [ml/m^2^]	Vvc [ml/m^2^]	Vvv [ml/m^2^]
(a) PA5%/Al.95%, filler fraction 0.4–0.8 mm	7.36	183.2	244.0	21.84
(b) PA10%/Al.90%, filler fraction 0.4–0.8 mm	0.92	39.6	23.93	18.42
(c) PA15%/Al.85%, filler fraction 0.4–0.8 mm	4.89	159.9	194.7	23.68
(d) PA5%/Al.95%, filler fraction 0.8–1.2 mm	1.723	142.1	132.5	22.83
(e) PA10%/Al.90%, filler fraction 0.8–1.2 mm	1.12	77.65	54.51	22.71
(f) PA15%/Al.85%, filler fraction 0.8–1.2 mm	1.09	23.27	21.19	20.55

**Table 4:** The volumetric composition of the composite after tomographic tests.

Type of Composite Sample	Metallic Filler [%]	Polymer Matrix [%]	Voids [%]	Contamination [%]	Uniformity of Composition [%]
(a) PA5%/Al.95%, filler fraction 0.4–0.8 mm	89.12	8.94	1.85	0.07	moderate
(b) PA10%/Al.90%, filler fraction 0.4–0.8 mm	78.85	21.04	0.07	0.04	v.high
(c) PA15%/Al.85%, filler fraction 0.4–0.8 mm	70.11	27.64	2.2	0.05	v.low
(d) PA5%/Al.95%, filler fraction 0.8–1.2 mm	88.50	9.95	1.5	0.05	low
(e) PA10%/Al.90%, filler fraction 0.8–1.2 mm	78.47	21.24	0.25	0.04	high
(f) PA15%/Al.85%, filler fraction 0.8–1.2 mm	69.65	29.04	1.3	0.05	low

## Data Availability

Data are contained within the article.

## References

[1] De Fazio D., Boccarusso L., Formisano A., Viscusi A., Durante M. (2023). A Review on the Recycling Technologies of Fibre-Reinforced Plastic (FRP) Materials Used in Industrial Fields. J. Mar. Sci. Eng..

[2] Jung H., Shin G., Kwak H., Hao T.M., Jegal J., Kim H.J., Jeon H., Park J., Oh D.X. (2023). Review of polymer technologies for improving the recycling and upcycling efficiency of plastic waste. Chemosphere.

[3] Balu R., Dutta N.K., Roy Choudhury N. (2022). Plastic Waste Upcycling: A Sustainable Solution for Waste Management, Product Development, and Circular Economy. Polymers.

[4] Kumar N.G., Rajesh K., Rama M., Durga Rao K.P., Bharath S., Manikanta J.E. (2023). A review on mechanical properties of hybrid polymer composites. Mater. Today Proc..

[5] Arunachalam S.J., Saravanan R. (2023). Study on filler reinforcement in polymer matrix composites—A review. Mater. Today Proc..

[6] Huseynov O., Hasanov S., Fidan I. (2023). Influence of the matrix material on the thermal properties of the short carbon fiber reinforced polymer composites manufactured by material extrusion. J. Manuf. Process..

[7] Pandit P.P., Liu C., Iacono S., Corti G., Hu Y. (2023). Microstructural Characterization and Property of Carbon Fiber Reinforced High-Density Polyethylene Composites Fabricated by Fused Deposition Modeling. Materials.

[8] Pinto G., Jiménez-Martín A. (2001). Conducting aluminium-filled nylon 6 composites. Polym. Compos..

[9] Osman A.F., Mariatti M. (2006). Properties of Aluminum Filled Polypropylene Composites. Polym. Polym. Compos..

[10] Schricker K., Bergmann J.P., Hopfeld M., Spie L. (2021). Effect of thermoplastic morphology on mechanical properties in laser-assisted joining of polyamide 6 with aluminum. Weld World.

[11] Dan-asabe B., Adeotio O., Samuel B.O. (2023). Development, characterization, and modeling of aluminum chips-gabbro filler polystyrene hybrid composite using mixture design. Mater. Chem. Phys..

[12] Anis A., Elnour A.Y., Alam M.A., Al-Zahrani S.M., AlFayez F., Bashir Z. (2020). Aluminum-Filled Amorphous-PET, a Composite Showing Simultaneous Increase in Modulus and Impact Resistance. Polymers.

[13] Martin M., Hanagud S., Thadhani N.N. (2007). Mechanical behavior of nickel+aluminum powder-reinforced epoxy composites. Mater. Sci. Eng. A.

[14] Bishay I.K., Abd-El-Messieh S.L., Mansour S.H. (2011). Electrical, mechanical and thermal properties of polyvinyl chloride composites filled with aluminium powder. Mater. Des..

[15] Suhas U., Shashidhara K.N., Bharath L. (2023). Mechanical Characterization of Copper and Aluminium Powder Reinforced Epoxy Polymer Composites. Int. J. Eng. Res. Technol..

[16] Alhamidi A., Anis A., Bashir Z., Alam M.A., Al-Zahrani S.M. (2023). Studies on the Effect of the Addition of Nano-Spherical Particles of Aluminum on the Thermal, Mechanical, and Morphological Properties of PBT–PET Blend Composites. Polymers.

[17] Dasture M.D., Kelkar D.S. (2007). Aluminium-filled low-density polyethylene structural, morphological, and mechanical properties. J. Appl. Polym. Sci..

[18] Ananth G., Smith R.A., Kumar A.A., Dakshna S., Harsath S. (2023). Fabrication of aluminium polymer composite. Int. J. Adv. Res. Innov. Ideas Educ..

[19] Zhou S., Hrymak A.N. (2024). Injection Molding of Polymers and Polymer Composites. Polymers.

[20] Xiao K.Q., Zhang L.C. (2002). The role of viscous deformation in the machining of polymers. Int. J. Mechancial Sci..

[21] Pierończyk J., Biało D. (2001). Selected problems of electrodischarge machining of aluminum matrix composites. Compos. Theory Pract..

[22] Mohit H., Rangappa M.S., Siengchin S., Gorbatyuk S., Manimaran P., Kumari C.A., Khan A., Doddamani M. (2022). A comprehensive review on performance and machinability of plant fiber polymer composites. Polym. Compos..

[23] Sheikh-Ahmad J., Davim J.P., Hocheng H. (2012). 5—Tool wear in machining processes for composites. Woodhead Publishing Series in Composites Science and Engineering, Machining Technology for Composite Materials.

[24] Ahmad J. (2009). Machining of Polymer Composites.

[25] Wang D., Onawumi P.Y., Ismail S.O., Dhakal H.N., Popov I., Silberschmidt V.V., Roy A. (2019). Machinability of natural-fibre-reinforced polymer composites: Conventional vs ultrasonically-assisted machining. Compos. Part A Appl. Sci. Manuf..

[26] Teti R. (2002). Machining of Composite Materials. CIRP Ann..

[27] Pecat O., Rentsch R., Brinksmeier E. Influence of Milling Process Parameters on the Surface Integrity of CFRP. Proceedings of the Fifth CIRP Conference on High Performance Cutting 2012.

[28] Han X., Xu D., Axinte D., Liao Z., Li H.N. (2021). On understanding the specific cutting mechanisms governing the workpiece surface integrity in metal matrix composites machining. J. Mater. Process. Technol..

[29] Dvořáčková Š., Kroisová D., Knápek T., Váňa M. (2024). Effect of Cutting Conditions on the Size of Dust Particles Generated during Milling of Carbon Fibre-Reinforced Composite Materials. Polymers.

[30] (2004). Aluminium and Aluminium Alloys—Chemical Composition and form of Wrought Products—Part 1: Numerical Designation System.

[31] (2018). Differential Scanning Calorimetry (DSC)—Part 3: De-Termination of Temperature and Enthalpy of Melting and Crystallization.

[32] (2019). Plastics—Determination of Dynamic Mechanical Properties—Part 1: General Principles.

[33] (2007). Tool shanks with 7/24 taper for automatic tool changers: Part 1: Dimensions and designation of shanks of forms A, AD, AF, U, UD and UF.

[34] Product Catalog, Hoffmann-Group. https://www.hoffmann-group.com/GB/en/houk/.

[35] (2021). Geometrical Product Specifications (GPS)—Surface Texture: Areal Part 2: Terms, Definitions and Surface Texture Parameters.

[36] Ratajczyk E. (2011). Tomografia komputerowa CT w zastosowaniach przemysłowych. Cz. I Idea pomiarów, główne zespoły i ich funkcje. Mechanik.

[37] Gnatowski A., Koszkul J. (2005). Influence of Soaking on Given Physical Properties and Structure of PA/PP Mixtures. J. Polym. Eng..

[38] Uematsu H., Kawasaki T., Koizumi K., Yamaguchi A., Sugihara S., Yamane M., Kawabe K., Ozaki Y., Tanoue S. (2021). Relationship between crystalline structure of polyamide 6 within carbon fibers and their mechanical properties studied using Micro-Raman spectroscopy. Polymer.

